# Myocardial Injury Predicts Risk of Short-Term All-Cause Mortality in Patients With COVID-19: A Dose–Response Meta-Analysis

**DOI:** 10.3389/fcvm.2022.850447

**Published:** 2022-05-02

**Authors:** Yuehua Li, Hanjun Pei, Chenghui Zhou, Ying Lou

**Affiliations:** ^1^Department of Cardiology, State Key Laboratory of Cardiovascular Disease, Fuwai Hospital, National Center for Cardiovascular Diseases, Chinese Academy of Medical Sciences and Peking Union Medical College, Beijing, China; ^2^Department of Cardiology, The First Affiliated Hospital of Baotou Medical College, Baotou, China; ^3^Department of Anesthesiology, State Key Laboratory of Cardiovascular Disease, Fuwai Hospital, National Center for Cardiovascular Diseases, Chinese Academy of Medical Sciences and Peking Union Medical College, Beijing, China

**Keywords:** cardiac troponin, myocardial injury, short-term mortality, meta-analysis, COVID-19

## Abstract

**Objective:**

Predictive value of myocardial injury as defined by elevated cardiac tropnins (cTns) in patients with COVID-19 has not been fully investigated. We performed a meta-analysis to evaluate the dose–response relationship between myocardial injury and short-term all-cause mortality.

**Methods:**

Pubmed, Embase, and the Cochrane Library database were searched for all the studies which evaluated the relationship between cTns and the risk of short-term all-cause mortality in patients with COVID-19.

**Results:**

Compared with patients without myocardial injury, the group with elevated cTns was associated with increased short-term mortality (11 studies, 29,128 subjects, OR 3.17, 95% CI 2.19–4.59, *P* = 0.000, *I*^2^ = 92.4%, *P* for heterogeneity 0.00). For the dose–response analysis, the elevation of cTns 1 × 99th percentile upper reference limit (URL) was associated with increased short-term mortality (OR 1.99, 95% CI 1.53–2.58, *P* = 0.000). The pooled OR of short-term mortality for each 1 × URL increment of cTns was 1.25 (95% CI 1.22–1.28, *P* = 0.000).

**Conclusion:**

We found a positive dose–response relationship between myocardial injury and the risk of short-term all-cause mortality, and propose elevation of cTns > 1 × 99th percentile URL was associated with the increased short-term risk of mortality.

## Introduction

COVID-19 pandemic caused by the SARS-COV-2 is responsible for an immense burden of morbidity and mortality globally. As of 13 December 2021, over 270.52 million confirmed cases have been identified and more than 5.32 million people died from COVID-19^1^. Patients affected by COVID-19 experience varied clinical presentations and outcomes. The majority of patients experience mild or moderate symptoms and resolve within a few weeks of initial infection, while other minorities develop acute respiratory distress syndrome (ARDS), or even multiple organ dysfunction with a high risk of mortality (1–3). The key element to reduce the mortality of COVID-19 has been recognized as identifying high-risk patients and providing earlier intervention (4, 5). However, it is still challenging to improve risk stratification.

According to the Fourth Universal Definition of Myocardial Infarction (UDMI), myocardial injury is defined as an increase of cardiac troponins (cTns, including cTnI and cTnT) values over the 99th percentile upper reference limit (URL). cTns, as a sensitive biomarker, could be detected not only in cardiac conditions, but also in non-cardiac conditions including sepsis, aortic dissection, end-stage renal disease, etc., (6–8). What's more, elevated cTns are often associated with adverse outcomes and are helpful for risk stratification in both cardiac and non-cardiac conditions (9–11). Till now, the predictive value of myocardial injury in patients with COVID-19 during the in-hospital term or short term is mixed. A series of studies have shown that myocardial injury in patients with COVID-19 is very common and associated with higher mortality (12–22). However, other studies indicated that the influence of myocardial injury is attenuated after adjusting multiple severe diseases (23, 24). Therefore, we performed a comprehensive dose–response meta-analysis to investigate the relationship between myocardial injury and short-term all-cause mortality in patients with COVID-19 with the following objectives: (1) to provide a quantitative assessment of the association between myocardial injury and short-term all-cause mortality; (2) to explore the potentially modifiable factors related to myocardial injury and short-term all-cause mortality; (3) to define an optimal threshold of elevated cTns that is associated mortality; (4) to quantify the dose–response relationship of the magnitude of myocardial injury and risk of short-term all-cause mortality.

## Methods

### Search Strategy

We reported this meta-analysis following the guidance of the MOOSE (Meta-analysis of Observational Studies in Epidemiology) statement (25). We searched PubMed (from 2019 to December 2012), Embase (from 2019 to December 2012), and the Cochrane Library database (http://www.cochrane.org). We also manually searched reference lists of the retrieved articles and reviews. The keywords used in the search were (troponin or myocardial injury) paired with (COVID-19). No language restriction was applied.

### Study Outcomes and Selection

The primary endpoint was short-term all-cause mortality. Inclusion criteria for the retrieved studies were as follows: (1) prospective or retrospective design; (2) inclusion of the outcome of short-term/in-hospital mortality; (3) inclusion of multivariable-adjusted or undusted relative risk (RR) or odds ratio (OR) and their corresponding 95% CI or provided the number of events and total population in each group; (4) inclusion of different level of elevated cTns and the related mortality. To conduct a dose–response meta-analysis, studies with three or more categories of URL were included (studies with <3°, e.g., with only positive or negative cTns were excluded); (5) the referent group with cTns <99th percentile URL or provided the number of events and cases within cTns <99th percentile URL.

### Data Extraction

Data were extracted by two independent authors (Yuehua Li and Hanjun Pei). Discrepancies were resolved by group discussion. The extracted data included the source of study (author, publication year, country), population characteristics [mean age, male proportion, number of subjects, percentage of elevated cTns, hypertension, diabetes mellitus (DM), coronary artery diseases (CADs), heart failure (HF), chronic obstructive pulmonary disease (COPD), chronic kidney disease (CKD), cancer], follow-up term, the different threshold of cTns, ORs, or RRs and their corresponding 95% CI. We assessed study quality by the Newcastle–Ottawa quality assessment scale which is a validated scale for non-randomized studies in meta-analyses. This scale assigns a maximum of nine points to each study: four points for selection, two points for comparability, and three points for the assessment of outcomes and adequacy of follow-up. We assigned scores of 0–3, 4–7, and 8–9 for low-, moderate-, and high-quality studies, respectively.

### Statistical Analysis

We considered RRs as ORs in the retrospective studies. We pooled the ORs from the group with the lowest URL and >99th percentile of URL in each study. If the study did not provide the ORs, we calculated the ORs by the number of events and total subjects in the non-elevated and elevated group. If different reference categories were reported, we chose a category with cTns <99th percentile of URL as reference. We pooled the OR by combining all the categories of elevated cTns for comparing the category with cTns ≥ 99th and <99th percentile of URL by DerSimonian and Laird random-effects model (26). If the study provided more than 3 categories, we would calculate the ORs using data on the number of cases and non-cases in all the elevated categories and referent groups. The random-effects model was also used in the pooled analysis for the potential clinical heterogeneity. The heterogeneity was assessed by *Q* statistic, *I*-squared, and *P*-value (*P* < 0.05 was considered to be statistically significant). Univariable meta-regression analyses (including all population characteristics, such as follow-up term, age, percentage of male, DM, hypertension, CAD, HF, COPD, CKD, cancer, and NOES points) were conducted to explore the potential sources of heterogeneity (27). For the dose–response analyses, the degree of elevated cTns was categorized into <99th percentile of URL, 1–2 URL, 2–3 URL, 3–5 URL, >5 URL. If the study provided the elevated cTns by numerical value, we converted it into the corresponding URL according to the upper reference value in each study. We assigned the ORs from each study into standardized intervals according to the range or median of the degrees of elevated cTns in each category. The average URL of elevated cTns in each category was estimated by mean of the lower and upper levels. If the highest category of cTns had an open upper level, the mean URL was estimated to be 1.5 level of the lower level. The weighted linear regression model was used to explore the dose–response relationship between elevated cTns and the risk of short-term all-cause mortality (28). To determine whether cTns was an independent risk factor for the short-term all-cause mortality, we performed sensitivity analysis restricted to the studies with multiple-variable adjusted OR.

Publication bias was assessed by the Begg's test and Egger's test. Two-sided *P* < 0.05 was considered to be significant (29). All the data analyses were performed by STATA software (10.0 version, StataCorporation, TX, USA) and REVMAN software (version 5.0; Cochrane Collaboration, Oxford, United Kingdom).

## Results

### Search Results

**Figure 1:**
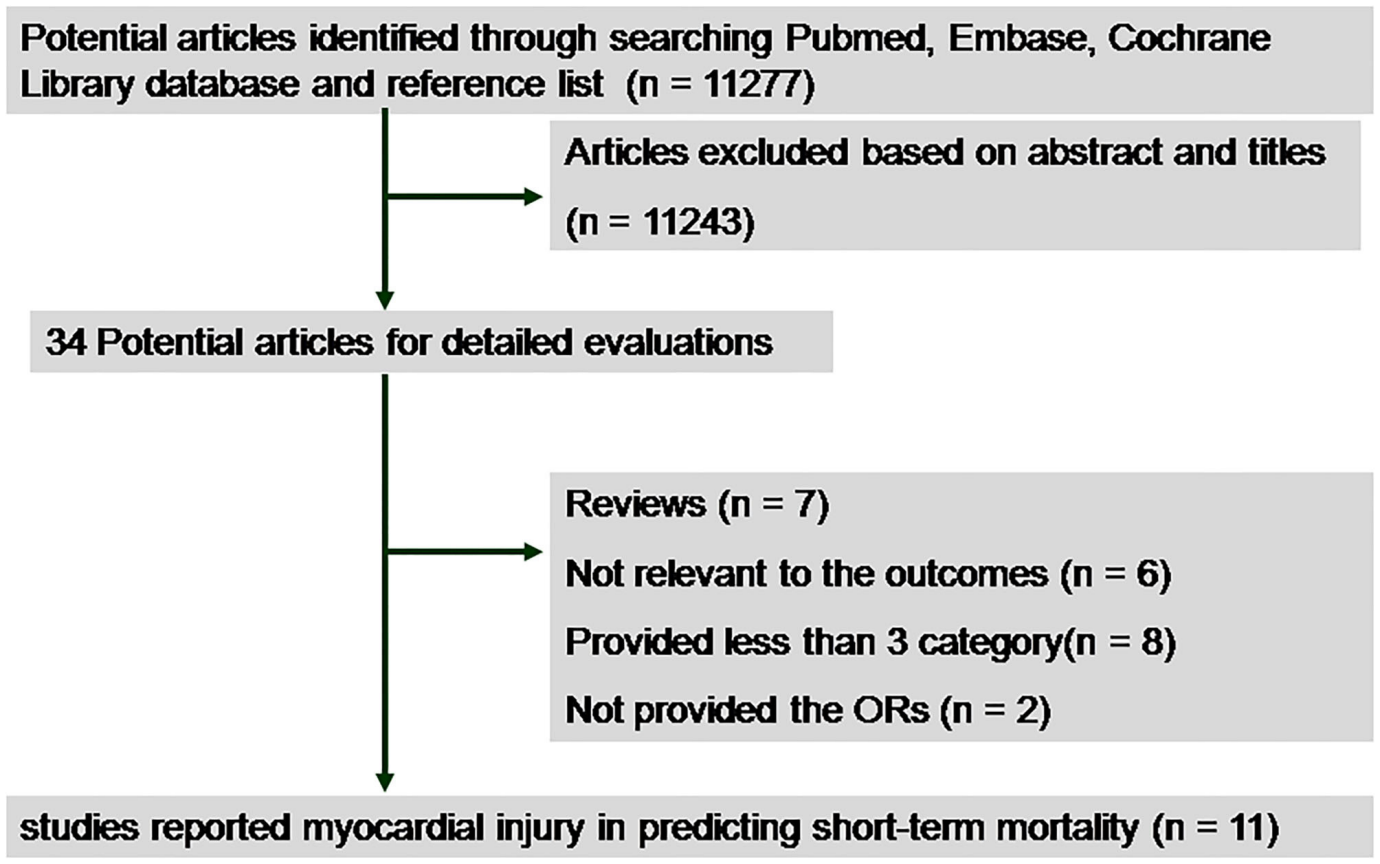
Flow chart of the trial selection process. OR, odds ratio.

We initially identified 11,277 studies by database and manual searching. After the exclusion of duplicates and non-relevant studies, 34 potential articles were selected for detailed evaluation. We further excluded 23 articles as shown in Figure 1. Finally, 11 retrospective studies involving 29,128 subjects were included in our meta-analysis (12–16, 18–23). Among them, nine had reported outcomes for cTnI (12, 14–16, 18, 20–23), 1 for cTnT (19), and 1 for combined cTnI and T (13).

### Study Characteristics

Table 1 showed the main characteristics of the data extracted from the included studies. All the studies had a retrospective study design. A total of eight studies were conducted in the USA and three in other countries. The mean age ranged from 49.0 to 68.0 years old, the follow-up time varied from 7 to 40 days. The incidence of myocardial injury in patients with COVID-19 ranged from 14.9 to 63.5%. The NOES points ranged from 6 to 9 (Supplementary Figure 1).

Table 1Characteristics of included studies.

**Table:** 

**References**	**Country**	**Category**	**Subjects**	**cTnI/T**	**Age (y)**	**Male %**	**Factory**	**Follow-up term**
Salvatici et al. (15)	Italy	< -99th %URL, 1–2 × 99th URL, >2 × 99th %URL	523	hs-cTnI	68.0	64	Beckman Coulter	7d
Almeida Junior et al. (19)	Brazi	≤0.006 ng/dl, 0.007–0.01 ng/dl, 0.011–0.029 ng/dl, ≥0.03 ng/dl	183	hs-cTnT	66.8	65.6	Roche Laboratory	7d
Franks et al. (20)	USA	< -99^th^%URL, 1-3 × 99^th^ URL,> 3 × 99^th^ %URL	182	cTnI	64.0	56.6	Abbott Architect i2000	In-hospital
Lala et al. (18)	USA	< -99th %URL, 1–3 × 99th %URL, > 3 × 99th %URL	2,736	cTnI	66.4	59.6	Abbott Architect i2001	14d
Metkus et al. (23)	USA	<99th %URL, 1–5 × 99th %URL, 5–10 × 99th % URL, >10 × 99th %URL	243	cTnI	62.8	60.9	Abbott Architect i2002	40d
Majure et al. (13)	USA	<99th %URL, 1–3 × 99th %URL, >3 × 99th %URL	6,247	cTnI, T	66.0	60	Siemens, Roche	7d
Raad et al. (16)	USA	<99th %URL, 1–5.5 × 99th %URL, >5.5 × 99th %URL	1,020	hs-cTnI	63.0	50	Beckman Coulter	30d
Tanboga et al. (12)	Turkey	<0.5 × 99th %URL, <99th %URL, 1–2 × 99th %URL, 2–5 × 99th %URL, 5–10 × 99th %URL, 10–50 × 99th % URL, >50 × 99th %URL	14,855	hs-cTnI	49.0	54	Abbott Architect i2000	30d
Chorin et al. (21)	USA	<99th %URL, 1–2 × 99th %URL, >2 × 99th %URL	204	hs-cTnI	64.0	76	Abbott Park	24.2 ± 7.4d
Smilowitz et al. (14)	USA	<99th %URL, 1–2.1 × 99th %URL, >2.1 × 99th %URL	2,163	hs-cTnI	64.1	63.3	Siemens, Abbot Architect	in-hospital
Ruge et al. (22)	USA	<99th %URL, 1–2 × 99th %URL, >2 × 99th %URL	772	cTnI	58.3	59.1	NA	in-hospital

**Table:** 

**References**	**HT %**	**DM %**	**CAD %**	**HF %**	**Cancer %**	**CKD %**	**COPD %**	**cTn(+) %**	**Adjusted variable**
Salvaticia et al. (15)	NA	NA	NA	NA	NA	NA	NA	NA	NA
Almeida Junior et al. (19)	53.6	19.7	19.1	NA	9.8	2.2	NA	63.5	Age, CAD, oxygen saturation, lymphocytes, D-Dimer, CRP, creatinine, BNP
Franks et al. (20)	NA	NA	NA	NA	NA	NA	NA	55.9	NA
Lala et al. (18)	38.9	10.1	16.6	10.1	7.1	10	5.8	36	Age, sex, BMI, race, ethnicity, history of CAD, history of AF, HF, HT, CKD, DM, statin use, ACEI or ARB use, and CURB-65 score
Metkus et al. (23)	60.9	19.2	NA	28.8	NA	20.2	22.2	51	Age, sex, creatinine, bilirubin, Pao2/FIo2 ratio, vasopressor use, lactate, organ failures
Majure et al. (13)	60	36	13	9	7	NA	6	29.2	Age, sex, race, ethnicity, HT, CAD, HF, peripheral vascular disease, COPD, and DM, use of ACEI/ARBs, alanine aminotransferase, and creatinine
Raad et al. (16)	73	44	12	13	NA	30	10	38.2	Age, sex, BMI, HT, CAD, Heart Failure, AF, cerebrovascular disease, COPD, CKD, cirrhosis, immunosuppressed state
Tanboga et al. (12)	36.3	19.9	15.3	5.1	3.1	3.2	21.6	6.9	Age, sex, NLR, D-Dimer, LDH, CRP, hemoglobin, platelet count, CAD, HF, COPD, cerebrovascular disease, HT, DM, CKD
Chorin et al. (21)	56	30	12	3		8	6	41	Age, CKD, DM, gender, race, CAD, HF, HT, COPD, HF, creatinine, abnormal LFTs
Smilowitz et al. (14)	NA	NA	NA	NA	NA	NA	NA	30.7	Age, sex, race, BMI, smoking, HT, hyperlipidemia, DM, CKD, previous myocardial infarction, HF, AF or malignancy, temperature, pulse oximetry at presentation, outpatient prescriptions for antiplatelets, statin and β-blocker use, CRP, creatinine, D-dimer, absolute lymphocyte count, and platelet count
Ruge et al. (22)	64.2	45.2	28	NA	11.3	14.8	8.2	14.9	NA

*ACEI, angiotensin-converting enzyme inhibitor; AF, atrial fibrillation; ARB, angiotensin II receptor blocker; BMI, body mass index; BNP, brain natriuretic peptide; CAD, coronary artery disease; CKD, chronic kidney disease; COPD, chronic obstructive pulmonary disease; CRP, C-reactive protein; cTn, cardiac troponin; hs-cTn, hypersensitive –cTn; DM, diabetes mellitus; HF, heart failure; HT, hypertension; LDH, lactate dehydrogenase; LFT, liver function test; NA, not available; NLR, neutrophil-lymphocyte ratio*.

### Myocardial Injury and Risk of Short-Term All-Cause Mortality

**Figure 2:**
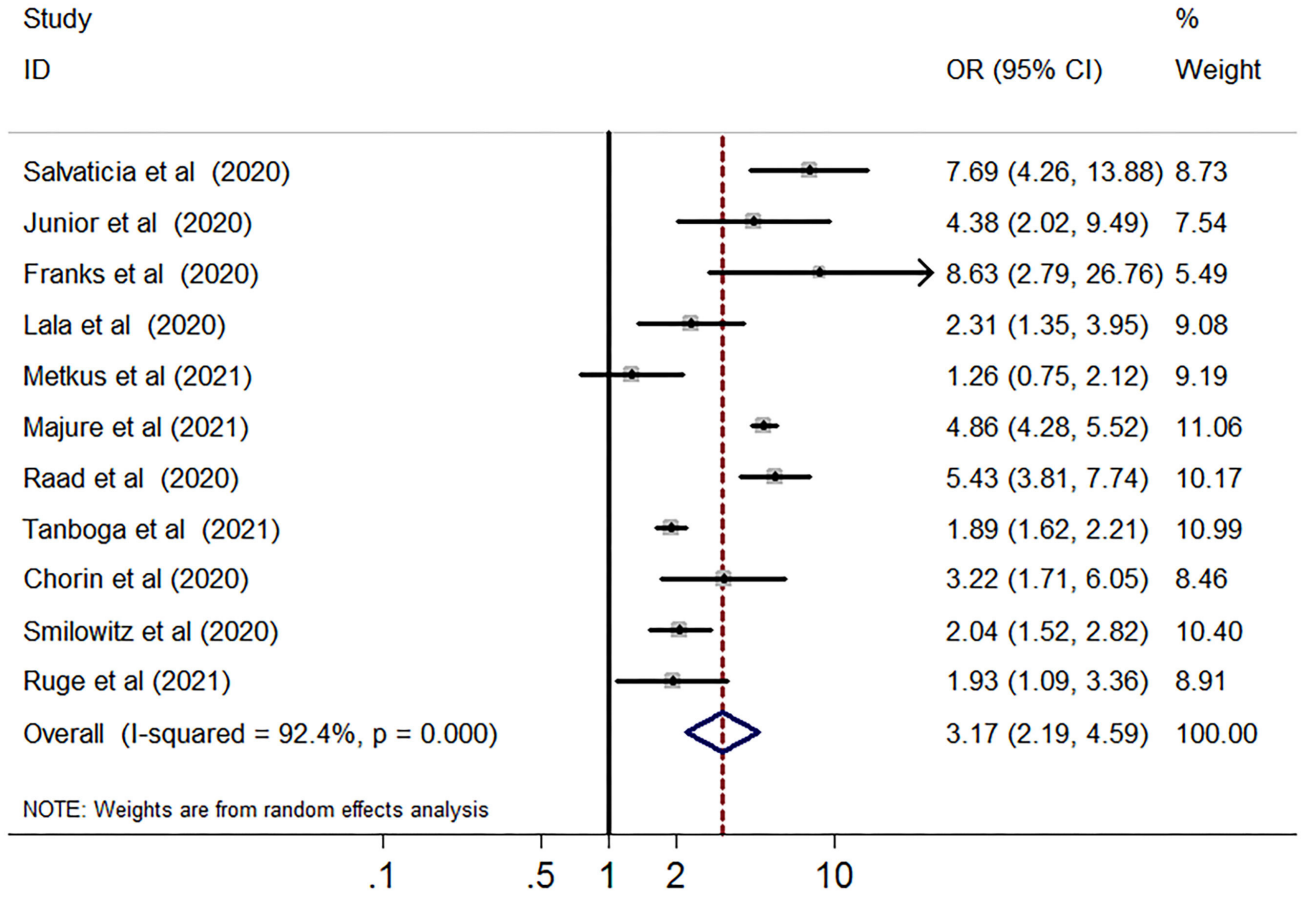
Funnel plot of myocardial injury and risk of short-term all-cause mortality in patients with COVID-19. Meta-analysis of elevated vs. non-elevated cardiac troponin levels and risk of short-term all-cause mortality in patients with COVID-19. CI, confidence interval; OR, odds ratio.

Compared with the group without myocardial injury, the group with myocardial injury was associated with an increased risk of short-term mortality (29,128 subjects, 11 studies, OR 3.17, 95% CI 2.19–4.59, *P* = 0.000, *I*^2^ = 92.4%, *P* for heterogeneity 0.00) in patients with COVID-19 (Figure 2). In univariable meta-regression, none of the variables including follow-up term, gender, age, percentage of DM, hypertension, CHD, HF, COPD, CKD, cancer, and NOES points was related to the risk of short-term death (Supplementary Table 2).

### Dose–Response Analysis of Myocardial Injury and Risk of Short-Term All-Cause Mortality

**Figure 3:**
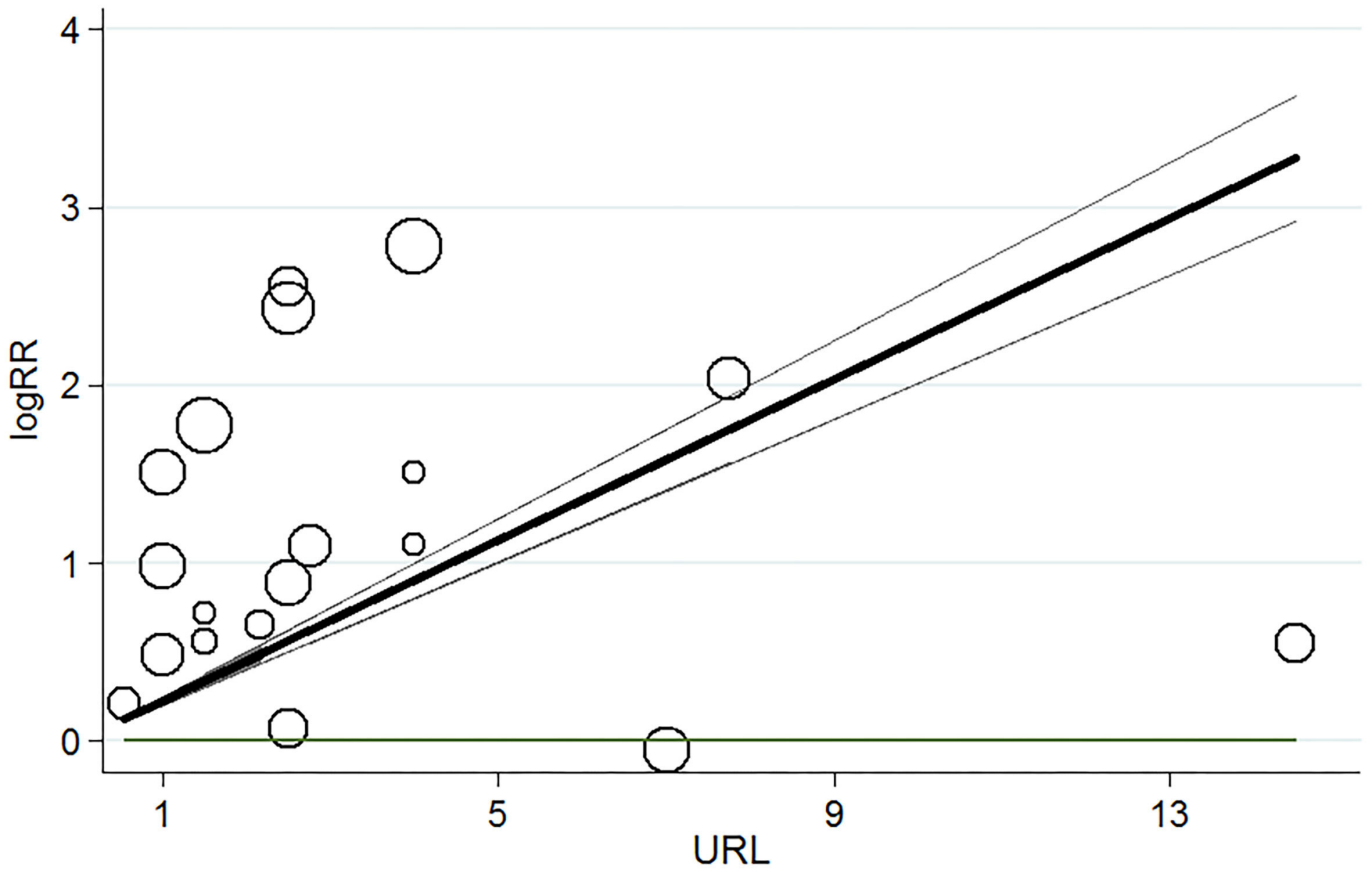
Dose–response relationship for myocardial injury and risk of short-term all-cause mortality in patients with COVID-19. Each black small circle indicates logOR for each category of cardiac troponin levels which is proportional to its statistical weight; solid line represents weighted logOR, and it is two accompanying dashed lines represent its lower and upper CIs. Horizonal solid line indicates the null hypothesis (logOR = 0). CI, confidence interval; OR, odds ratio.

Table 2 showed the elevated cTns > 99th percentile URL was associated with increased risk of short-term mortality in patients with COVID-19 (OR 1.99, 95% CI 1.53–2.58, *P* = 0.000, *I*^2^ = 62.3%, *P* for heterogeneity 0.007). The dose–response analysis showed that for every 1x99^th^ percentile URL increment in cTns elevation, the pooled OR was 1.25 (95% CI 1.22–1.28, *P* = 0.000) for the risk of all-cause mortality in patients with COVID-19 (Figure 3).

**Table 2:** Risk of short-term mortality by categories of cardiac troponin I in patients with COVID-19.

**Category (URL)**	**No. of studies**	**OR 95% CI**	***P***	***P* for heterogeneity**
1 to <2x	9	1.96 (1.53–2.58)	0.000	0.007
≥2 to <3x	9	2.92 (1.97–4.33)	0.000	0.000
≥ 3x to <5x	5	3.45 (2.27–5.22)	0.000	0.000
≥ 5x	4	2.48 (1.09–5.67)	0.000	2.93

*CI, confidence interval; OR, odds ratio; URL, upper reference limit*.

### Sensitivity Analysis

When restricted to studies with the multivariable-adjusted results [13–15, 17, 19, 22–24), we found that cTns was also associated with the short-term death (eight studies, 28,240 subjects, OR 2.09, 95% CI 1.72–2.53, *P* = 0.000, *I*^2^ = 33.8%, *P* for heterogeneity 0.158) (Supplementary Figure 1). The dose–response analysis showed that for every 1 × 99th percentile URL increment in cTns elevation, the pooled OR was 1.23(95% CI 1.20–1.26, *P* = 0.000) for the risk of short-term all-cause mortality in patients with COVID-19 (Supplementary Figure 2).

### Publication Bias

Publication bias was not observed by Begg's adjusted rank correlation test (*P* = 0.436) and Egger's test (*P* = 0.832) (Supplementary Figure 3).

## Discussion

In this dose–response meta-analysis of retrospective studies, we found that myocardial injury as defined by elevated cTns above the 99th percentile URL was associated with a 3.17-fold increased risk of short-term mortality in patients with COVID-19. This association was not modified by the factors including age, gender, follow-up term, percentage of DM, hypertension, CAD, HF, COPD, CKD, and cancer. More importantly, above 1 × 99th percentile URL for cTns was related to the risk of short-term mortality. Crucially, the short-term mortality was increased by 25% for each 1 × 99th percentile URL increment for cTns.

Accumulating evidence has indicated that myocardial injury is very common in patients with COVID-19 and often predicts poor prognosis. Bangalore et al. (30) have reported that in a case series with ST-segment elevation affected by COVID-19, 9 of 10 patients with myocardial injury died in the hospital even without coronary involvement, compared with the group with confirmed myocardial infarction deemed less death (4/8). Shi et al. (31) have shown that higher cTnI predicted in-hospital death in severe patients with COVID-19 [hazard ratio (HR) 4.26] and the author also reported that the predictive value of myocardial injury ranged during the time from symptom onset (HR, 4.26) and from admission to endpoint (HR 3.41) (32). Our study was in line with the previous researches and showing that myocardial injury was associated with the short-term mortality. What's more, our meta-analysis has provided new insights. Our study has provided a cut-off value for myocardial injury and indicated that elevated cTns above the 1 × 99th percentile URL were associated with the risk of short-term mortality. Of note, our dose–response analysis also revealed a positive linear association between myocardial injury and short-term mortality in patients with COVID-19 (OR 1.25). Therefore, it would be helpful to risk-stratify the patients with COVID-19 by routine screening for cTns at admission.

The association of myocardial injury and risk the short-term all-cause mortality might be modified by combined diseases such as cardiovascular diseases, respiratory disease, kidney disease, or malignancy. Zhou et al. (33) have indicated that myocardial injury was associated with in-hospital mortality in unadjusted model and the predicted value was attenuated after adjusted age, gender, current smoking, combined diseases including DM, hypertension, CAD, lung diseases, kidney diseases, and inflammatory factors. However, Shi et al. (32) have reported that myocardial injury is an independent risk factor for the risk of mortality after adjusting the mentioned variables. Our meta-regression analyses were consistent with the latter study and showed that the predictive value of myocardial injury was not modified by the mentioned factors. What is more, our sensitivity analysis restricted to the multiple-variable adjusted studies has also shown the positive dose–response relationship between cTns and short-term death. Our results suggested that myocardial injury was an independent risk factor for short-term mortality in patients with COVID-19. Nevertheless, large prospective trials are necessary to investigate the modifiable factor for myocardial injury.

The present meta-analysis has important strengths. First, our meta-analysis indicated that myocardial injury is an independent risk factor for short-term mortality in patients with COVID-19. Our meta-analysis suggested that routine screening cTns at admission would be helpful for risk-stratification and guide further management for patients with COVID-19. Second, we have provided a cut-off value for elevated cTns about the risk of short-term mortality, that is cTns above the 1 × 99th percentile URL. Using this cut-off value, patients at a higher risk of death could be identified. Last but not the least, our dose–response results have shown that each increment of 1 × 99th percentile URL, results in increase in short-term mortality by 25%. The patients with a high level of cTns should be paid attention and may benefit from a prolonged hospital stay, closer monitoring, more intensified treatment, or more intensive outpatient follow-up to improve outcomes. Future research to aim at preventing or reducing the development of myocardial injury warrants further investigation, given the dose–response between cTns release and adverse outcomes.

The present meta-analysis also has some limitations. First, our meta-analysis was based on retrospective studies, so the recalling and selective bias might be a concern. Second, not all studies have provided the multi-variable adjusted ORs, so the residual confounders could not be ruled out. However, we performed sensitivity analysis restricted to the studies with multi-variable adjusted and found that there was also a positive dose–response relationship between cTns and short-term death in patients with COVID-19. Third, we have excluded some studies without providing more than three categories of cTns, so the unpooled data might affect the results. Fourth, heterogeneity is often a concern of meta-analysis. We tried to explore the potential heterogeneity but were limited by other data such as cardiac function, heart rate, respiratory rate, atrial fibrillation, cough, lung involvement by CT scanning, etc. Fifth, the potential different blood sampling regimens for cTns levels may result in some inherent heterogeneity. Sixth, although the two tests showed no obvious publication bias, we could not rule out the potential effect on the results. Seventh, for few studies, have provided the results of cTn above 10 × 99th percentile URL, thereby the dose–response relationship in a higher level of cTn is limited. Finally, our meta-analysis used pooled data, rather than individual data, which restricted the potential confounding factors.

## Conclusion

Our dose–response meta-analysis of 11 studies comprising 29,128 patients with COVID-19 has demonstrated that myocardial injury was an independent risk factor for the risk of short-term mortality. We provided the optimal cut-off value of myocardial injury which is the 99th percentile URL about short-term mortality. Each increment of 1 × 99th percentile URL of cTns, the short-term mortality was increased by 25%. Routine screening of cTns at admission is helpful to risk stratification and to guide therapy for patients with COVID-19.

## Data Availability Statement

The original contributions presented in the study are included in the article/Supplementary Material, further inquiries can be directed to the corresponding authors.

## Author Contributions

CZ conceived the idea and supervised the study. YL and HP acquired and analyzed the data and took the responsibility for the accuracy and integrity of the data. YL and YL drafted the manuscript. CZ, YL, YL, and HP contributed to writing, reviewing, or revising the article. All authors contributed to the article and approved the submitted version.

## Funding

This work was supported by the National Natural Science Foundation of China (nos. 81970290 and 81760096), PUMC Youth Fund, and Fundamental Research Funds for the Central Universities (no. 2017320009).

## Conflict of Interest

The authors declare that the research was conducted in the absence of any commercial or financial relationships that could be construed as a potential conflict of interest.

## Publisher's Note

All claims expressed in this article are solely those of the authors and do not necessarily represent those of their affiliated organizations, or those of the publisher, the editors and the reviewers. Any product that may be evaluated in this article, or claim that may be made by its manufacturer, is not guaranteed or endorsed by the publisher.

## Footnotes

^1^ https://www.worldometers.info/coronavirus/

## Supplementary Material

Supplementary Figure 1 Funnel plot of myocardial injury and risk of short-term all-cause mortality in COVID-19 patients. Meta-analysis of elevated vs. non-elevated cardiac troponin levels and risk of short-term all-cause mortality in COVID-19 patients for studies with multiple-variable adjusted results. CI, confidence interval; OR, odds ratio.

Supplementary Figure 2 Dose-response relationship for myocardial injury and risk of short-term all-cause mortality in COVID-19 patients for studies with multiple-variable adjusted results. Each black small circle indicates logOR for each category of cardiac troponin levels which is proportional to its statistical weight; solid line represents weighted logOR, and its two accompanying dashed lines represent its lower and upper CIs. Horizonal solid line indicates the null hypothesis (logOR = 0). CI, confidence interval; OR, odds ratio.

Supplementary Figure 3 Begg's funnel plot (with pseudo 95% CIs) of myocardial injury and risk of short-term all-cause mortality with all individual studies. Studies that evaluated the association of myocardial injury and risk of short-term all-cause mortality were plotted with weighted lnOR on the vertical axis and the se of the lnOR along the horizontal axis. CI, confidence interval; OR, odds ratio; SE, standard error.

Supplementary Table 1 Summarized Newcastle–Ottawa quality assessment scale of the included randomized trials.

Supplementary Table 2 Meta-regression of baseline characteristics for elevated cardiac troponin and risk of short-term all-cause mortality for COVID-19 patients.
