# Supplementary figures and images for: Myocardial Injury Predicts Risk of Short-Term All-Cause Mortality in Patients With COVID-19: A Dose–Response Meta-Analysis
Source: Front Cardiovasc Med. 2022 May 2;9:850447. doi: 10.3389/fcvm.2022.850447 (PMC9108210; doi:10.3389/fcvm.2022.850447)

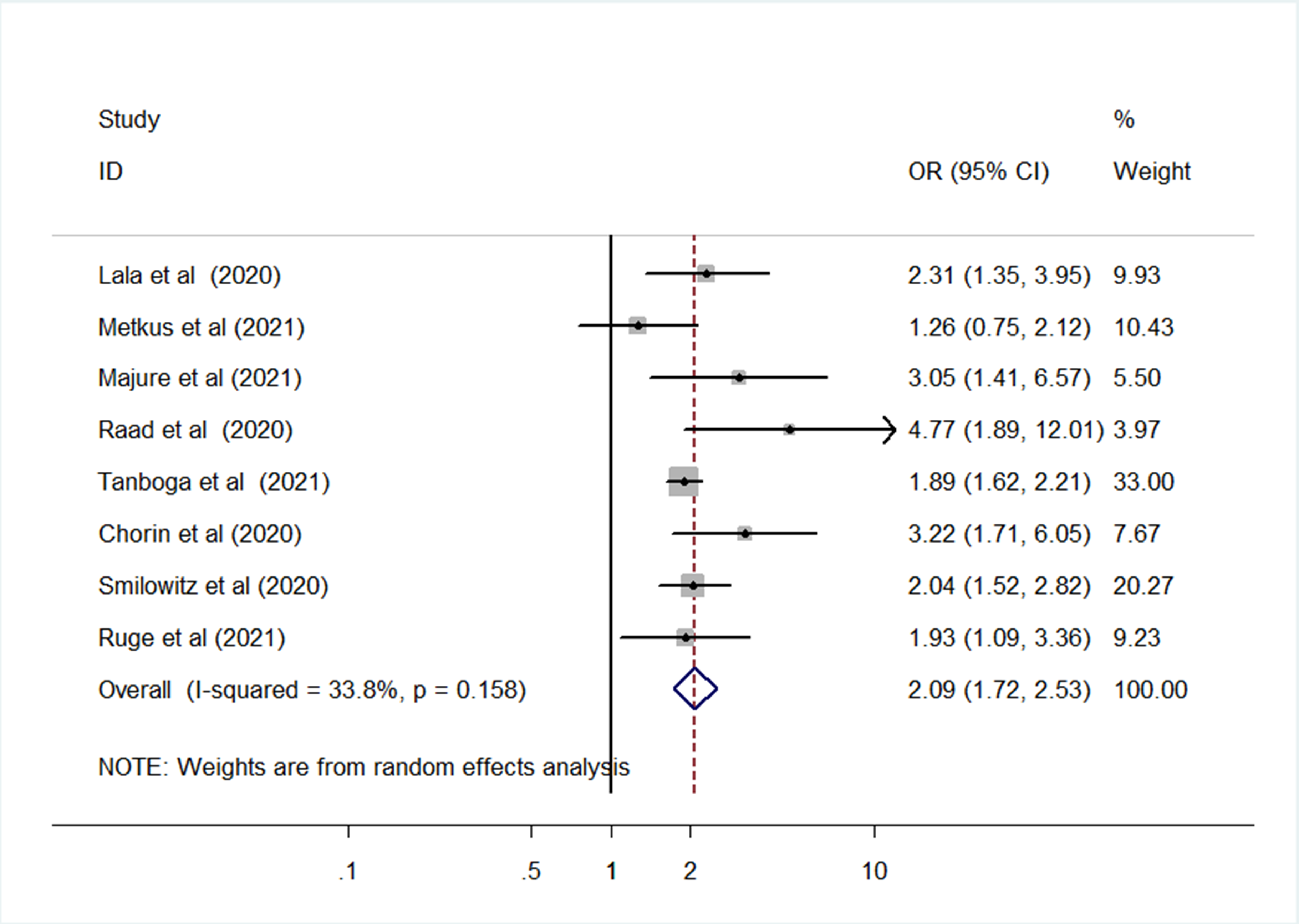

Supplement: Supplementary Figure 1 — Funnel plot of myocardial injury and risk of short-term all-cause mortality in COVID-19 patients. Meta-analysis of elevated vs. non-elevated cardiac troponin levels and risk of short-term all-cause mortality in COVID-19 patients for studies with multiple-variable adjusted results. CI, confidence interval; OR, odds ratio. [file Image_1.TIF]

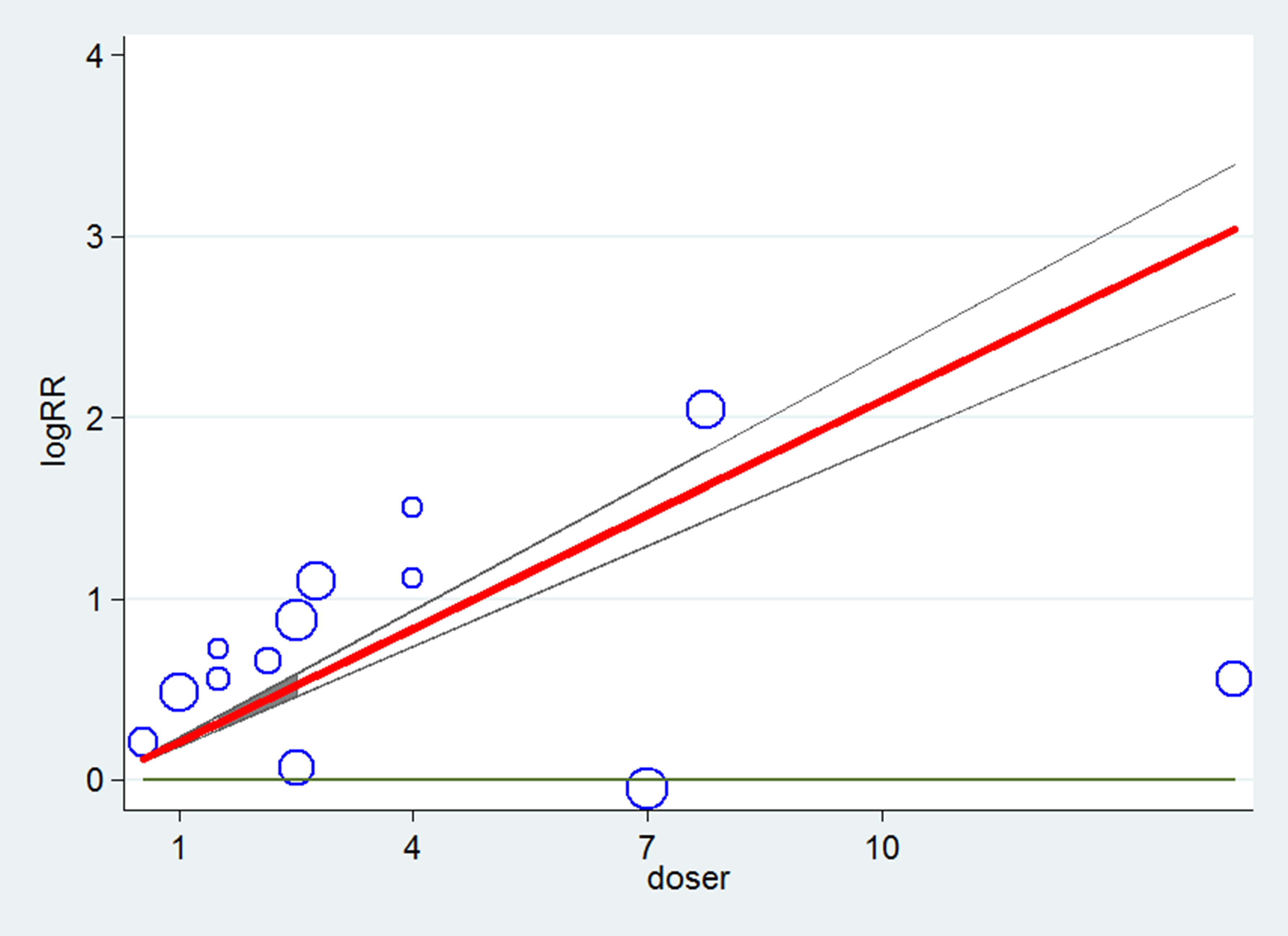

Supplement: Supplementary Figure 2 — Dose-response relationship for myocardial injury and risk of short-term all-cause mortality in COVID-19 patients for studies with multiple-variable adjusted results. Each black small circle indicates logOR for each category of cardiac troponin levels which is proportional to its statistical weight; solid line represents weighted logOR, and its two accompanying dashed lines represent its lower and upper CIs. Horizonal solid line indicates the null hypothesis (logOR = 0). CI, confidence interval; OR, odds ratio. [file Image_2.TIF]

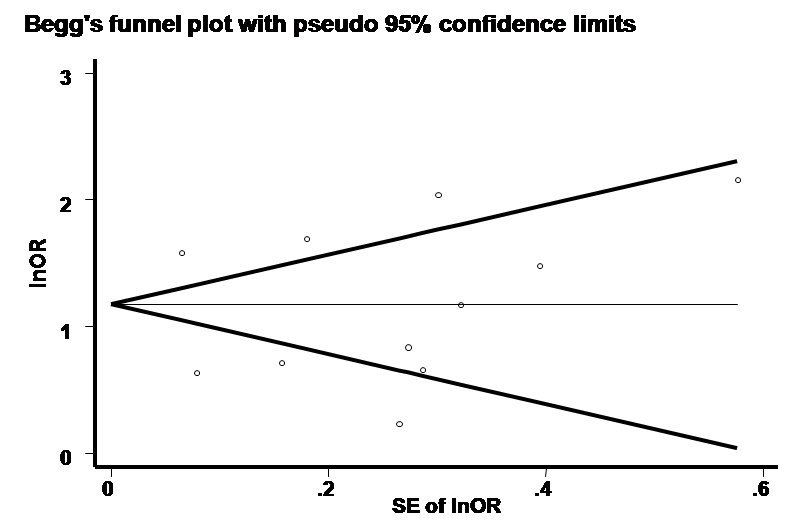

Supplement: Supplementary Figure 3 — Begg's funnel plot (with pseudo 95% CIs) of myocardial injury and risk of short-term all-cause mortality with all individual studies. Studies that evaluated the association of myocardial injury and risk of short-term all-cause mortality were plotted with weighted lnOR on the vertical axis and the se of the lnOR along the horizontal axis. CI, confidence interval; OR, odds ratio; SE, standard error. [file Image_3.TIF]
