# Supplementary material for: Myocardial Injury Predicts Risk of Short-Term All-Cause Mortality in Patients With COVID-19: A Dose–Response Meta-Analysis
Source: Front Cardiovasc Med. 2022 May 2;9:850447. doi: 10.3389/fcvm.2022.850447 (PMC9108210; doi:10.3389/fcvm.2022.850447)
Supplement: Supplementary Table 1 — Summarized Newcastle–Ottawa quality assessment scale of the included randomized trials. [file Table_1.pdf]

Supplementary Table 1 Summarized Newcastle–Ottawa quality assessment scale of the included randomized trials

Study	Patient selection	Comparability	Exposure	Total Score
Chorin 2020	2	2	3	7
Franks 2020	1	2	3	6
Junior 2020	2	2	3	7
Lala 2020	4	2	3	9
Raad 2020	3	2	3	8
Salvatici 2020	4	2	3	9
Smilowitz 2020	3	2	3	8
Majure 2020	3	2	3	8
Metkus 2020	4	2	3	9
Tanbo˘ga 2020	3	2	3	8
Ruge 2020	3	2	3	8
