# Supplementary material for: Myocardial Injury Predicts Risk of Short-Term All-Cause Mortality in Patients With COVID-19: A Dose–Response Meta-Analysis
Source: Front Cardiovasc Med. 2022 May 2;9:850447. doi: 10.3389/fcvm.2022.850447 (PMC9108210; doi:10.3389/fcvm.2022.850447)
Supplement: Supplementary Table 2 — Meta-regression of baseline characteristics for elevated cardiac troponin and risk of short-term all-cause mortality for COVID-19 patients. [file Table_2.pdf]

Supplementary Table 2 meta-regression of baseline characteristics for elevated cardiac troponin and risk of short-term all-cause mortality for COVID-19 patients

Baseline characteristics	Number of trials	Risk of short-term all-cause mortality			
		Coefficient	95% CI	I^2 (%)	P
Age	11	0.053	(-0.017to 0.122)	83.27	0.120
Male proportion	11	-0.003	(-0.071 to 0.006)	92.58	0.936
Percentage of hypertension	8	0.017	(-0.010 to 0.055)	84.38	0.297
Percentage of diabetes	8	0.020	(-0.016 to 0.057)	82.40	0.226
Percentage of coronary artery disease	7	-0.048	(-0.134 to 0.039)	92.20	0.216
Percentage of heart failure	6	-0.028	(-0.109 to 0.053)	96.21	0.387
Percentage of cancer	5	0.032	(-0. 241 to 0.304)	92.67	0.736
Percentage of chronic kidney disease	7	0.012	(-0.449 to 0.069)	76.59	0.614
Percentage of COPD	7	-0.048	(-0.112 to 0.015)	77.06	0.108
Follow-up term	10	-0.019	(-0.042 to 0.005)	86.71	0.101
NOES point	10	-0.263	(-0.777 to 0.252)	92.93	0.278

CI: confidence interval; COPD: chronic obstructive pulmonary disease, NOES: Newcastle–Ottawa quality assessment scale
